# The influence of sports on proactive personality and academic achievement of college students: The role of self-efficacy

**DOI:** 10.3389/fpsyg.2022.943347

**Published:** 2022-08-31

**Authors:** Xinnan Li, Min Liu, Haofei Yu, Zhanjia Zhang, Zhonghui He

**Affiliations:** ^1^School of Physical Education and Art Education, Beijing Technology and Business University, Beijing, China; ^2^School of Physical Education and Sports Science, Qufu Normal University, Qufu, China; ^3^Physical Education (PE) Department, Renmin University of China, Beijing, China; ^4^The Department of Physical Education, Peking University, Beijing, China

**Keywords:** different sports group, proactive personality, self-efficacy, academic performance, college students

## Abstract

**Objective:**

To explore the mediating effect of self-efficacy on the relationship between the proactive personality and academic performance of college students in different sports groups.

**Methods:**

A questionnaire survey is used to study 552 college students. The research tools include the proactive personality scale, the self-efficacy scale table (general, academic, and self-regulation efficacy scale tables), and the academic performance self-report scale table. This research employs SPSS 11.0 statistical software to carry out correlation analysis, regression analysis, and *t*-tests on the data collected, while the test of mediating effect is carried out by AMOS 22.0.

**Results:**

(1) The degree of self-efficacy and academic performance of college students participating in physical activities is significantly greater than that of the non-sports group; (2) the proactive personality level of the sports group is significantly higher than the non-sports group in the dimension of “conscientiousness”; (3) a confirmatory factor analysis of the mediating effect hypothesis model, using the structural equation model, found that self-efficacy plays a full mediating role in the relationship between proactive personality and academic performance; the direct effect of self-efficacy on the proactive personality and academic performance of college students in different sports groups is not significant.

**Conclusion:**

College students involved in sports exercise have higher scores on some items about proactive personality than non-sports groups; girls’ self-efficacy level is higher than that of boys; self-efficacy plays a full mediating role in the relationship between proactive personality and academic performance; self-efficacy had no significant effect on proactive personality and gender in different sports groups.

## Introduction

Current sports at home and abroad attach great importance to the level of physical activity of students, because physical activity is effective in promoting the development of physical and mental health of college students, which is necessary for their healthy growth and for the development of national training of healthy talents (Su and Liu, 2019). The Chinese health physical education curriculum model focuses on the fact that contemporary students should improve their physical health and mental health to form a healthy lifestyle and develop positive psychological qualities, which are important concepts for higher education to help contemporary college students cultivate physical and mental health. Positive psychological qualities have an important role in promoting the development of good physical and mental health of college students, and proactive personality is an important quality. Proactive personality is a stable individual variable that improves performance by actively manipulating the environment in which it is placed (Thomas et al., 1993). Individuals need to act proactively to change the behavioral tendencies of the external environment, and the proactive personality of college students has an important predictive role for academic performance and has a close relationship with self-efficacy (Johan et al., 2009). Numerous studies have shown that proactive personality has a positive impact on college students’ career, employment motivation, academic self-efficacy, adaptability, and learning ability (Shang and Gan, 2009; Zhao, 2013), and is closely related to college students’ study efficiency, life habits, and mental health level, which are important indicators of them having a healthy personality and positive psychological qualities. Individuals with high levels of proactive personality have positive attitudes and behaviors toward environmental adaptation and are able to adopt a proactive approach to cope with stress and frustration, thus enhancing self-efficacy in work and study. Proactive personality has an important influence on individual self-efficacy, which is manifested in student employment, career decision-making, adaptability, and job performance. Therefore, revealing the influencing factors of college students’ proactive personality and its pathways of action, and providing scientific methods and means for the comprehensive development of college students are of great significance to their academic performance improvement and healthy physical and mental development.

Western psychology has long been devoted to exploring the factors influencing the formation of proactive personality traits. Based on years of research, researchers have proposed numerous theories about proactive personality. The most representative theories are the active motivation processing model, and the drive model proposed by researchers (Sharon et al., 2010). Based on these two models, a theoretical model about proactive personality and academic efficacy and achievement has been put forward, and after confirmatory factor analysis, the model showed that college students’ academic self-efficacy plays a fully mediating role between proactive personality and academic performance (Wang and Wang, 2016). There is a significant positive correlation between proactive personality, general self-efficacy, and academic adaptation (Li, 2014). Chinese scholars explored the relationship between proactive personality and sports, and the results showed that athletes with high proactive personality have more autonomy when participating in sports (Ai and Wang, 2017). Athletes with high proactive personalities actively adjust their coping styles in stressful situations, transforming stress into motivation and mobilizing themselves to adapt to the high-intensity competition environment and atmosphere. The theoretical model of exercise behavior (Mao et al., 2013) suggests that by understanding the high and low level of one’s proactive personality, the possibility of improving it through sports is greater. Using the influence of self-efficacy on the academic performance of different groups of proactive personalities can effectively help college students form a proactive mentality to achieve good grades, develop good exercise habits, and cultivate a healthy lifestyle and study attitude, thus developing a healthy, well-rounded body and mind, which is also a type of “virtuous circle.” Studies on the correlation between exercise and self-efficacy show that moderate-intensity aerobic exercise contributes to the improvement of self-efficacy and of the mental health of college students (Liu et al., 2007), and that long-term regular exercise can promote self-efficacy and self-confidence. Research on the cognitive function of exercise on college students showed that moderate-intensity exercise can improve college students’ memory capacity, thus contributing to their academic level (Li et al., 2013). In summary, there are strong relationships between sports and proactive personality, self-efficacy, and academic performance. Specifically, while exercise has an effect on proactive personality and academic performance, its level of self-efficacy is not a complete and direct predictor of academic performance without participation in exercise. Self-efficacy may play a bridging role. Therefore, this study attempts to propose the hypothesis that self-efficacy plays a mediating role in the effect of proactive personality on academic performance across sports groups.

The studies that have been conducted to explore the relationships between sports, self-efficacy, proactive personality, and academic performance are mostly descriptive and univariate analyses. There are mainly the following aspects in the studies that need to be explored in depth: First, there is a lack of in-depth exploration of the mechanisms that influence exercise to improve academic performance, and many of the findings can only indicate that there is a correlation between exercise and academic performance. However, there is not yet sufficient evidence for a causal relationship between the two, and even less exploration involving any mediating or moderating variables between them, so strict control of confounding variables and scientifically rigorous experimental studies designed to establish a causal relationship are needed. Second, the differences between the groups of research subjects have not been highlighted. In particular, the differences between males and females have been the key point in psychological research work. Studying the strengths and weaknesses of men and women in different areas, revealing the differences of sports on academic performance and its influencing factors between different genders, and giving appropriate education and guidance for both sexes, are of far-reaching significance for the good development of their physical and mental health and the improvement of their academic life.

**FIGURE 1:**
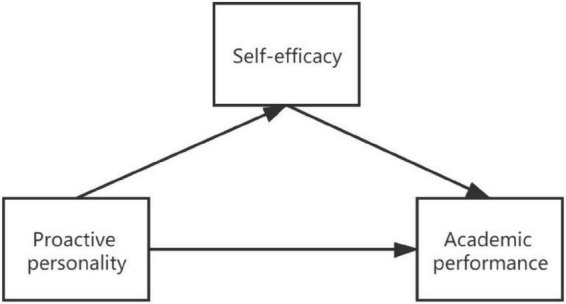
Model of the relationships between proactive personality, self-efficacy, and academic performance.

In summary, based on active motivation processing, drive model theory, and the exercise behavior theoretical model, combined with existing empirical studies, we put forward the hypothesis of “active personality → self-efficacy → academic performance” (see Figure 1), aiming to theoretically understand in depth the impact of the level of proactive personality of different sports groups on academic performance mediated by self-efficacy. Practically, we provide interventions and guidance to improve academic performance and promote the physical and mental health of individuals.

## Research methods

### Study population

Using class-based cluster sampling, a questionnaire survey was conducted among 580 current first-year and sophomore students at a comprehensive university in Beijing, and 552 valid questionnaires were returned, with an effective rate of 95%. The mean age of the survey respondents was 20.12 years (*SD* = 1.58); 201 were male and 351 were female. Among them, there were 296 first-year students and 256 sophomores; 261 arts students and 291 science students; 278 in the sports group and 274 in the non-sports group.

### Measurement tools

#### Proactive personality scale

The Proactive Personality Scale, as revised by Li (2014), was used to fit Chinese university students. The original scale was developed by Thomas et al. (1993). The scale has 11 items in three dimensions—resilience, changeability, and responsibility—and is scored on a five-point Likert scale, from 1 “not at all” to 5 “fully,” with higher scores indicating a more positive proactive personality. The scale has been widely used in China and has good reliability and validity. In the study, the internal consistency alpha coefficient of the scale was 0.95.

#### Comprehensive self-efficacy scale

The Comprehensive Self-Efficacy Scale consists of three subscales: the General Self-Efficacy Scale, the Academic Self-Efficacy Scale (ASES), and the Self-Regulation Efficacy Scale. All three subscales are scored on a five-point Likert scale (from 1 “not at all” to 5 “fully”), and the higher the score on each subscale, the higher the level of self-efficacy. Scholars in China translated the Chinese version of the scale, examined its structure, reliability, and validity, and found that all three subscales could be applied to the measurement of self-efficacy of Chinese people, and that all three subscales had high reliability and validity.

##### General self-efficacy scale

General self-efficacy, as a stable personality trait, is a general level of confidence in an individual’s ability to cope with new or difficult situations (Sherer et al., 1982; Schwarzer et al., 1997; Chen et al., 2000). The General Self-Efficacy Scale (GSES) was developed in 1981 by Dan Ralf Schwarzer, a German clinical psychologist and health psychologist, and his colleagues. The Chinese version used was the General Self-Efficacy Scale translated by Wang (2001), which has 10 items and is the most widely used scale to measure non-domain-specific self-efficacy. The internal consistency alpha coefficient of this questionnaire in the study was 0.98.

##### Academic self-efficacy scale

Academic self-efficacy is a subjective feeling and judgment on whether an individual has the self-confidence and ability to complete a task in the learning domain; it is expressed as the self-confidence of an individual to correctly assess his or her own ability (Bandura, 1977). The scale is mainly used to measure self-efficacy in the academic domain regarding general learning ability with 12 items. The internal consistency alpha coefficient of this questionnaire in the study was 0.98.

##### Self-regulation efficacy scale

Self-regulation is a process by which an individual consciously and systematically directs his or her thinking, feelings, and behaviors to achieve a specific goal (Bandura, 1977). Self-regulated learning is a learning style in which an individual uses metacognitive and other cognitive strategies to actively engage in learning. Self-regulation also requires the use of school-based programs to empower individuals to be self-motivated (Zimmerman, 2000). Bandura states that the ability to choose appropriate learning strategies and to know how to self-regulate based on one’s current level is self-regulated learning. The Self-Regulation Efficacy Scale, based on Bandura’s Child Self-Efficacy Scale and the Self-Regulated Learning Efficacy Subscale, is a nine-item scale that measures the self-efficacy of an individual’s self-regulated behavior in learning. The internal consistency coefficient of the questionnaire in this study was 0.98.

#### Assessment of academic performance

A self-reported approach was used to measure college students’ academic performance, in which college students were asked to rate their academic performance on a seven-point scale, with higher ratings indicating better academic performance (Zhang et al., 2011).

### Definition of sports groups

In this study, the sports group was defined as those who participated in exercise two or more times a week for 30–60 min each time, besides physical education classes. Those who cannot meet this characteristic are included in the non-sports group (Yin et al., 2007; Sun, 2009).

### Statistical analysis

SPSS 21.0 software was used for statistical analysis of the data. Statistical methods such as independent samples *t*-test, correlation analysis, analysis of variance, and structural equation modeling were used to analyze the differences in demographic variables in terms of proactive personality, self-efficacy, and academic performance, as well as the relationships between them among college students. AMOS 22.0 was used to test for mediating effects, and a mediating effect model was developed and validated based on the theory of Wen et al. (2004). Also, two variables—sports/non-sports groups, and gender—were used to distinguish different groups and test for differences between different groups.

## Results and analysis

### The change of college students’ proactive personality in different sports groups and their gender differences

In order to explore whether the proactive personality of college students in different sports groups changes because of sports, and whether there is gender difference in the different proactive personality, taking proactive personality as the dependent variable and different students’ sports groups and gender as the independent variable, the analysis of variance between two-way ANOVA was conducted (see Tables 1, 2).

**TABLE 1:** The average proactive personality of different genders and different sports groups.

Gender	Student groups	*M*	*SD*	*n*
Male	Sports group	37.56	8.41	119
	Non-sports group	39.92	7.45	84
	Total	38.54	8.09	203
Female	Sports group	42.52	9.62	159
	Non-sports group	39.82	9.65	190
	Total	41.05	9.72	349
Total	Sports group	40.40	9.43	278
	Non-sports group	39.85	9.02	274

**TABLE 2:** Results of the proactive personality ANOVA for the tracking accuracy.

Variable	Class III sum of squares	*Df*	Mean square	*F*	*P*
Gender	740.65	1	740.65	8.97	0.00**
Students group	3.66	1	3.66	0.04	083
Gender*students group	807.68	1	807.68	9.79	0.00**

**P ≤ 0.01, *P ≤ 0.05.

The results from Table 2 showed that: first, the gender main effect was significant, *F* = 8.97, *P* < 0.01; The main effect of group of students was no significant, *F* = 0.04, *P* > 0.05; There was significant interaction between gender and different student groups (*F* = 9.79, *P* < 0.01).

**FIGURE 2:**
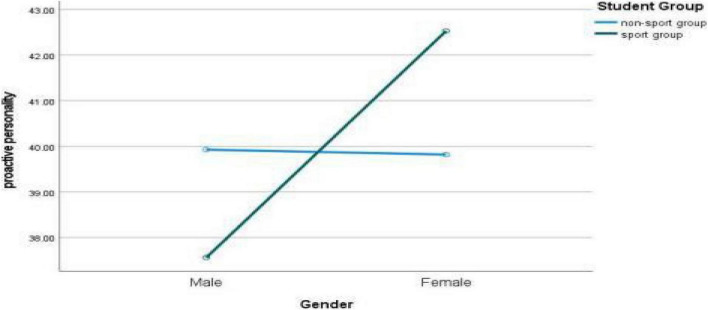
Gender difference of college students’ proactive personality in different sports groups.

The changes and gender differences of college students’ proactive personality in different sports groups can be seen in Figure 2.

### The change of college students’ academic achievement in different sports groups and their gender differences

In order to explore whether the academic achievement of college students in different sports groups changes because of sports, and whether there is gender difference in the different academic performance, taking academic performance as the dependent variable and different students’ sports groups and gender as the independent variable, the analysis of variance between two-way ANOVA was conducted (see Tables 3, 4).

**TABLE 3:** The average academic achievement of different genders and different sports groups.

Gender	Student groups	*M*	*SD*	*n*
Male	Sports group	3.98	0.73	119
	Non-sports group	3.81	1.24	84
	Total	3.91	0.974	203
Female	Sports group	4.28	1.04	159
	Non-sports group	4.13	0.77	190
	Total	4.20	0.90	349
Total	Sports group	4.15	0.93	278
	Non-sports group	4.03	0.95	274

**TABLE 4:** Results of the academic achievement ANOVA for the tracking accuracy.

Variable	Class III sum of squares	*Df*	Mean square	*F*	*P*
Gender	12.17	1	12.17	14.16	0.00**
Students group	3.15	1	3.15	3.67	0.05*
Gender*students group	0.01	1	0.01	0.01	0.92

**P ≤ 0.01, *P ≤ 0.05.

The results from Table 4 showed that: first, the gender main effect was significant, *F* = 14.16, *P* < 0.01; The main effect of group of students was significant, *F* = 3.15, *P* = 0.05; There was no significant interaction between gender and different student groups (*F* = 0.01, *P* > 0.05).

**FIGURE 3:**
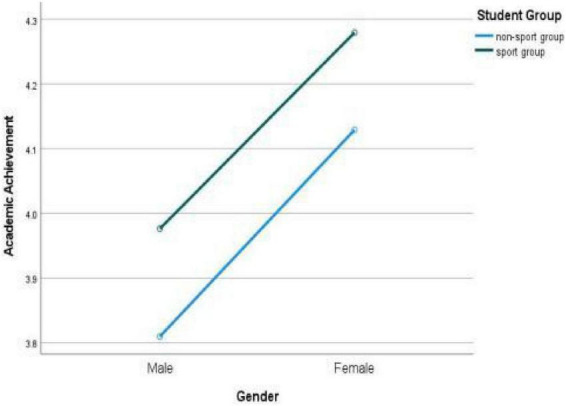
Gender difference of college students’ academic achievement in different sports groups.

The changes and gender differences of college students’ academic performance in different sports groups can be seen in Figure 3.

### The change of college students’ self-efficacy in different sports groups and their gender differences

In order to explore whether the self-efficacy of college students in different sports groups changes because of sports, and whether there is gender difference in the different self-efficacy, taking self-efficacy as the dependent variable and different students’ sports groups and gender as the independent variable, the analysis of variance between two-way ANOVA was conducted (see Tables 5, 6).

**TABLE 5:** The average self-efficacy of different genders and different sports groups.

Gender	Student groups	*M*	*SD*	*n*
Male	Sports group	36.92	5.61	119
	Non-sports group	36.13	5.92	84
	Total	36.60	5.74	203
Female	Sports group	35.43	5.42	159
	Non-sports group	35.96	4.42	190
	Total	35.72	0.90	349
Total	Sports group Non-sports group	36.07	5.54	278
		36.01	4.92	274

**TABLE 6:** Results of the self-efficacy ANOVA for the tracking accuracy.

Variable	Class III sum of squares	*Df*	Mean square	*F*	*P*
Gender	86.75	1	86.74	3.17	0.07
Students group	2.09	1	2.09	0.07	0.78
Gender*students group	55.27	1	55.27	2.02	0.15

*P ≤ 0.05.

The results from Table 6 showed that: first, the gender main effect was no significant, *F* = 3.17, *P* > 0.05; The main effect of group of students was no significant, *F* = 0.07 *P* > 0.05; There was no significant interaction between gender and different student groups (*F* = 2.02, *P* > 0.05).

**FIGURE 4:**
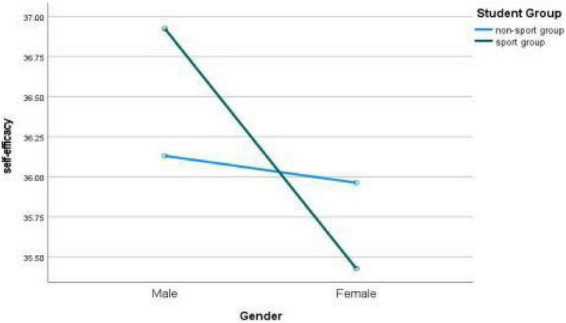
Gender difference of college students’ self-efficacy in different sports groups.

The changes and gender differences of college students’ self-efficacy in different sports groups can be seen in Figure 3. As can be seen from Figure 4, students’ self-efficacy has an interaction effect on different sports groups and genders, but the interaction effect is not significant, indicating that the self-efficacy of college students in sports groups and non-sports groups will change due to gender differences, but the change degree is not obvious.

### Relationships between proactive personality, self-efficacy, and academic performance

A Pearson’s correlation analysis was conducted on proactive personality, self-efficacy, and academic performance. The correlation coefficients between all the variables studied reached a significant level, except for the two dimensions of “resilience” and “changeability” in proactive personality and academic performance, which were not correlated (Table 7).

**TABLE 7:** Analysis of the correlation between proactive personality, self-efficacy, and academic performance.

Variable	1	2	3	4	5	6	7	8
1 Proactive personality	–							
2 Resilience	0.93**	–						
3 Changeability	0.93**	0.83**	–					
4 Responsibility	0.93**	0.77**	0.81**	–				
5 Comprehensive self-efficacy	0.16**	0.14**	0.12**	0.16**	–			
6 General self-efficacy	0.16**	0.15**	0.13**	0.16**	0.98**	–		
7 Academic self-efficacy	0.14**	0.14**	0.11**	0.14**	0.99**	0.96**	–	
8 Self-regulation efficacy	0.17**	0.14**	0.13**	0.19**	0.97**	0.93**	0.95**	–
9 Academic performance	0.09**	0.08	0.06	0.11**	0.88**	0.85**	0.88**	0.86**

**P ≤ 0.01.

### The mediating role of self-efficacy in proactive personality and academic performance among college students

As shown in Table 7, the academic performance of college students was significantly positively related to proactive personality and comprehensive self-efficacy. To examine the relationships between proactive personality, self-efficacy, and academic performance among college students, and to test the mediating role of self-efficacy, latent variable structural equation modeling was used to construct a model of the relationships between the three variables. In the structural equation model, academic performance was the observed variable, and proactive personality and self-efficacy were latent variables, containing multiple observed variables. The results showed that the model fit indices CFI, NFI, and IFI were all greater than 0.90, 2 < *X*^2^/_*df*_ < 3, and RMSEA < 0.08, indicating that the model fit well (Table 8; Li et al., 2010).

**TABLE 8:** Fit indices of the model of the relationships between proactive personality, self-efficacy, and academic performance among college students

Model	*X^2^/df*	GFI	NFI	IFI	CFI	RMESA
Research model	2.91	0.99	0.94	0.96	0.96	0.05

As shown by the path relationship diagram of the effect of proactive personality and self-efficacy on academic performance among college students (Figure 1), proactive personality did not have a direct and significant effect on academic performance (β = -0.05, *p* > 0.05), but did have a significant positive prediction on comprehensive self-efficacy (β = 0.39, *p* < 0.05); moreover, comprehensive self-efficacy had a significant positive prediction of academic performance (β = 0.4, *p* < 0.05), which indicates that comprehensive self-efficacy plays a fully mediating role in the effect of proactive personality on academic performance. The effect of college students’ proactive personality on academic performance would be fully mediated by comprehensive self-efficacy. Comprehensive self-efficacy had different predictive effects on three sub-efficacy and positive predictive effects on academic and general efficacy (β = 0.87, β = 0.75, *p* < 0.05), further indicating that academic and general efficacy play a significant role in the effect of comprehensive self-efficacy on academic performance, while self-regulation efficacy in this module did not play a dominant role. Proactive personality had a significant effect on academic performance through the mediating role of self-efficacy, and among the three subdimensions of proactive personality, changeability, and resilience, they had a positive predictive effect (β = 0.93, β = 0.89, *p* < 0.05), further indicating that resilience and changeability are two important influences in the effect of proactive personality on comprehensive self-efficacy. With respect to self-regulation efficacy in comprehensive self-efficacy, and responsibility in proactive personality, there was a correlation between the parts of these two dimensions that could not be explained by their own latent variables. The two dimensions that could not be explained were partially correlated.

**FIGURE 5:**
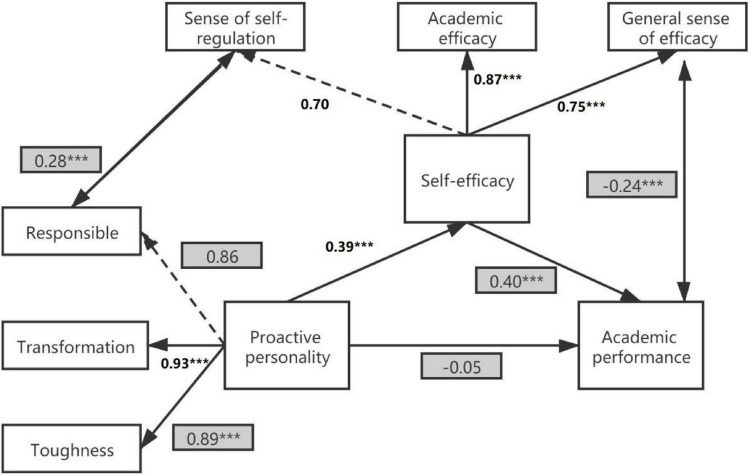
Model of the relationships between proactive personality, self-efficacy, and academic performance among college students. ***P < 0.001.

As shown in Figure 5, the direct effect of proactive personality on academic performance was -0.05, the total effect was 0.12, and the indirect effect was 0.16; the direct effect of proactive personality on comprehensive self-efficacy was 0.39, the total effect was 0.39, and the indirect effect was 0.00; and the direct effect of comprehensive self-efficacy on academic performance was 0.4, the total effect was 0.4, and the indirect effect was 0.00.

### Analysis of group differences in the relationships between proactive personality, self-efficacy, and academic performance in different sports groups

The mediated effects models were first tested separately for the sports group and the non-sports group, establishing the unrestricted model (M1) and the model with equal structural coefficients (M2); the results of the two model fits are shown in Table 4. The results indicate that the above multiple models fit the data well, with RMSEAs = 0.04 < 0.5 and GFI = 0.98. There was no significant difference between M1 and M2 (*p* = 0.26 > 0.05), indicating that there was no significant difference in the structural model coefficients between the sports and non-sports groups. As the holistic nature did not represent the between-group effect for specific factor loadings, other individual variables were examined through “parametric pairing.” After comparing the critical ratio values for parametric differences, it was found that there was a significant difference in the effect of changeability on proactive personality between the sports/non-sports groups (c.r = 2.05, *P* < 0.05), with an absolute value greater than 1.96; there was no significant difference in the effect of proactive personality on self-efficacy between different sports groups (c.r = 0.86, *P* > 0.05). There was no significant difference between different sports groups in the effect of proactive personality on academic performance; nor between different sports groups in the effect of academic self-efficacy on academic performance (c.r = 0.83, *P* > 0.05) (see Tables 9, 10).

**TABLE 9:** Comparison of different group models of the mediating effect of self-efficacy—different sports groups.

Model	*X* ^2^	*df*	RMSEA	GFI	TLI
M_1_	42.05	20	0.04	0.98	0.90
M_2_	49.23	26	0.04	0.97	0.91

**TABLE 10:** Decomposition of the effects of the mediated model of **self-efficacy for different sports groups**.

Impact pathways	Non-sports groups (Normalized path coefficient [standard error])	Sports groups (Normalized path coefficient [standard error])
Proactive personality → self-efficacy	0.32*** (0.10)	0.48*** (0.1)
Proactive personality → academic performance	–0.04 (–0.14)	–0.03 (0.02)
Self-efficacy → academic performance	0.42*** (0.17)	0.36*** (0.01)

***P < 0.001.

### Analysis of group differences by gender in the relationships between proactive personality, self-efficacy, and academic performance

The mediated effects models were tested separately for males and females, establishing the unrestricted model (M3) and the model with equal structural coefficients (M4); the results of the two model fits are shown in Table 6. The results show that the above multiple models fit the data well, with RMSEAs = 0.03 < 0.5 and GFI = 0.98. The lack of significant difference between M3 and M4 (*p* = 0.62 > 0.05) indicates that there is no significant difference between genders in measuring the structural model coefficients. As the holistic nature did not represent the between-group effect for specific factor loadings, other individual variables were examined through “parametric pairing.” After comparison of the critical ratio values for parametric differences, it was found that there was no gender difference in the effect of academic self-efficacy on self-efficacy (c.r = -0.08, absolute value less than 1.96, *p* > 0.05) and no gender difference in the effect of self-regulation efficacy on self-efficacy (c.r = -0.07, absolute value less than 1.96, *p* > 0.05). There was no gender difference in the effect of changeability on proactive personality (c.r = 0.83, absolute value less than 1.96, *p* > 0.05), no gender difference in the effect of resilience on proactive personality (c.r = 0.90, absolute value less than 1.96, *p* > 0.05), and no gender difference in the effect of proactive personality on self-efficacy (c.r = -0.5, absolute value less than 1.96, *p* > 0.05). The structural covariance model of *P* = 0.00 < 0.01 further revealed that there was a significant difference in covariance coefficients between male and female groups. After comparing the critical ratio values of parameter differences, we found that there was a significant covariance difference in the effect of proactive personality on self-efficacy between the two groups (c.r = 2.76, absolute value greater than 1.96, *P* < 0.05), indicating a significant covariation relationship between proactive personality and self-efficacy in both male and female groups (see Tables 11, 12).

**TABLE 11:** Comparison of different group models of mediating effects of self-efficacy—different gender groups.

Model	*X* ^2^	df	RMSEA	GFI	TLI
M_3_	71.96	30	0.03	0.98	0.97
M_4_	86.28	42	0.03	0.97	0.98

**TABLE 12:** Effect decomposition of the mediating model of self-efficacy for different gender groups.

Impact pathways	Male student [Normalized path coefficient (standard error)]	Female student [Normalized path coefficient (standard error)]
Proactive personality to self-efficacy	0.38*** (0.06)	0.35*** (0.1)
Proactive personality → academic performance	–0.03 (0.01)	0.00 (0.02)
Self-efficacy → academic performance	0.39*** (0.01)	0.26** (0.01)

***P < 0.001.

## Discussion

### Proactive personality, self-efficacy, and academic performance of college students and their differences in different demographic variables

The results of this study show that college students have a high level of proactive personality, with the dimension of “responsibility” being significantly higher in the sports group than in the non-sports group. Responsibility in proactive personality is mainly reflected in clear and organized goals, taking effective actions, proactively solving problems, following up tasks in an orderly manner according to a certain schedule, holding self-confidence in their abilities, and having a sense of responsibility (Li, 2014). Related studies show that college students who are proactive have the courage to face and believe in their ability to change whatever situation they encounter (Wang and Wang, 2016). The level of mental health of sports groups is significantly higher than that of non-sports groups, and those who regularly participate in sports are better than non-sports groups in active communication and coordination of interpersonal relationships (Yin et al., 2007). According to American sports sociologist Jay Coakley, organizational skills in sports activities are an important way to develop the ability of youth to have responsibility (Shi, 2018). College students who adhere to regular exercise not only have the ability to take the initiative to solve problems, but also have the ability to take positive action; in addition, the long-term adherence to exercise motivates them to plan their exercise time, intensity, and frequency, or to complete a sports project in a sports group, cultivating their responsibility to treat each exercise task with full dedication, to set an appropriate goal, and to complete it responsibly. In sports groups with a high proactive personality, the increased sense of responsibility drives them to be proactive in facing changes in their environment, and to plan and act for the setting and accomplishment of their own goals.

The level of self-efficacy of the sports group was significantly higher than that of the non-sports group, with the levels of three subdimensions of self-efficacy—general, academic, and self-regulation efficacy—being higher in the sports group than in the non-sports group. This indicates that the overall self-confidence of the regular sports group is higher than that of the infrequent sports group, especially in terms of confidence in self when dealing with difficulties, self-evaluation when encountering challenging things in learning, and positive regulation in the face of emotional frustration or self-perception. It has been shown that there are many mediating variables in the effect of sports on positive psychology that influence many behavioral outcomes. According to self-efficacy theory, its impact on human beings is important and universal; it influences motivation, emotions, and behavior; the way sports groups behave toward their own sports is autonomous; they are strongly motivated; no matter what type of sport they perform, their internal motivation in the process is stronger than that of non-sports groups; and most importantly, with continuous exercise, the increase in physical posture or skill level makes those in the sports group increasingly confident. This confidence will definitely transfer to life and learning, and the self-efficacy will be stronger due to the strengthening of positive emotional experiences with regular exercise (Zhou, 2004). Sports and non-sports groups differ in their external and implicit regulation of pleasurable emotions, with sports groups showing elevated pleasurable emotions, and non-sports groups showing reduced pleasurable emotions (Yin et al., 2015).

The academic performance of college students in sports groups is significantly higher than that in non-sports groups. College students’ academic performance reflects their academic ability, in which memory ability plays a crucial role as an important factor in learning ability to improve academic performance. Studies related to physical activity and learning memory ability show that regular exercise training improves learning memory function (Yang et al., 2009). Exercise increases the release of nerve growth factor, which plays a key role in promoting learning memory through exercise. This suggests that college students in sports groups who maintain regular exercise can stimulate factors in their brains that improve learning memory capacity, and their academic performance will improve compared to non-sports groups.

Girls had significantly higher levels of self-efficacy than boys, with significantly higher levels of general self-efficacy and self-regulation efficacy than boys. Girls scored higher than boys on the overall self-efficacy competencies, with no significant difference in academic self-efficacy, mainly demonstrated in the levels of general self-efficacy and self-regulation efficacy. This indicates that girls are more confident than boys in their self-efficacy and self-regulation abilities in the face of adversity. Some studies have shown that male students have higher levels of tension, anger, fatigue, high energy, and self-esteem than female students (Yin et al., 2007), which would indicate that compared to the female group, male students’ self-efficacy are affected by many aspects, and their self-confidence in facing self-selection and cognitive dilemmas is disturbed by more factors. Self-efficacy of female college students is negatively correlated with compulsions, interpersonal relationships, and anxiety in mental health (Liu et al., 2007), and research in the field of mental health has shown that female college students are more emotionally susceptible and volatile (Bradley et al., 2001; Shields, 2003). This shows that female students’ general self-efficacy and self-regulation efficacy are affected by fewer factors, and their level of efficacy can be enhanced when they can control their own emotions.

### The effect of proactive personality on academic performance: The mediating role of self-efficacy

The study found that proactive personality has a significant positive effect on self-efficacy, while self-efficacy has a significant positive effect on academic performance. Thus, self-efficacy plays a fully mediating role between proactive personality and academic performance. This result concurs with existing studies (Wang and Wang, 2016). In self-efficacy, general self-efficacy and academic self-efficacy are more significant for academic performance, while at the same time, the resilience and changeability nature of proactive personality have more significant effects on self-efficacy. Proactive personality continues to play an important role in the learning process of students, which affects the level of self-efficacy, as reflected in their general self-efficacy and their level of self-confidence when facing academic problems. This shows that students with proactive personality can adapt and change to their environment and to motivate themselves to believe that they can overcome difficulties when facing any dilemma. They take the initiative to overcome problems and create better conditions to improve their academic performance (Sharon et al., 2010).

The study also found a correlation between self-regulation efficacy and “responsibility” in proactive personality. General self-efficacy and academic performance, two components that cannot be explained by one’s own latent variables, are correlated. General self-efficacy is a measure of a person’s overall self-confidence in a task that he or she may not be professionally trained for, but feels confident in completing the task; he or she can also be confident that he or she can overcome academic challenges, cross-discipline, and other disadvantages. This group with high general self-efficacy may not complete these tasks, especially academic tasks, but they are still confident when they encounter a gap in their knowledge. The academic performance of such groups probably depends in large part on an initial encouragement of self, which comes from their own self-confidence and ability. This part of the study may be more reflected in new environments, or in groups with high potential. This will play a supporting role in talent development and the selection of outstanding teams.

Self-regulation efficacy is understood at a cognitive level as an ability that is difficult to possess, and most studies on the effect of self-efficacy on academic performance directly examine academic efficacy and its encompassing learning abilities and behavioral efficacy. However, with the development and change of the actual situation, academic self-efficacy affecting academic performance cannot be a single dominant factor, especially for different groups of college students who need to have the ability to self-regulate their sense of efficacy. This is because it requires students to have an objective assessment of their own level, to adopt certain learning strategies in the learning process and, more importantly, to be able to guide their own learning ability. When an individual has a negative learning attitude, individuals with high self-regulation efficacy will calm their emotions through positive cues to the self, recover from the negative state to a smooth emotion, and finally guide their self-learning strategies into a positive emotional state.

Individuals with proactive personalities have long-term goals for the future, and in achieving this goal, they adjust their approach and strategies according to the actual situation, but they do not change their self-affirmation and can rise to the occasion in the face of difficulties, until significant changes occur (Sharon et al., 2010).

### Differences in the mediating role of self-efficacy across sports groups and gender

The study revealed no sports group differences regarding the mediating role of self-efficacy in the relationship between proactive personality and academic performance. There were no gender differences in the mediating role of self-efficacy in the relationship between proactive personality and academic performance.

The effect of self-efficacy as a mediator in the effect of proactive personality on academic performance across sports and gender groups was not significant. The absence of correlation between physical exercise and general self-efficacy of college students suggests that there is no effect of proactive personality on academic performance as moderated by self-efficacy in sports vs. non-sports groups and males vs. females. This is further explained by the fact that college students in the sports group do not improve their academic performance, due to their high levels self-efficacy and proactive personality. In contrast, students in the non-sports group do not have decreased academic performance, due to low levels of proactive personality and self-efficacy. A related study showed that there was no significant difference between physical activity on the general self-efficacy of male and female college students (Zhang and Sun, 2009); this finding is consistent with the fact that self-efficacy of different sports groups in this study was not mediated among male and female students. This differs from the hypothesis and is an issue to be further studied. Perhaps because there are many influences on academic performance, of which self-efficacy and sports are only two, the relationship between sports and proactive personality is more complex, with many factors influencing proactive personality, and with many unknown moderating variables between the two. However, it is worth establishing that the proactive personality–self-efficacy–academic performance pathway is significant, and that proactive personality, self-efficacy, and academic performance are higher in the sports group than in the non-sports group. Other variables that moderate differences between sports groups cannot be ignored in the process of proactive personality influencing academic performance, and need to be explored further.

## Conclusion

(1) There are significant group differences in the level of “responsibility” among college students of the sports/non-sports groups, with the sports group scoring significantly higher than the non-sports group; there are significant group differences in the level of self-efficacy among college students in these groups, with the sports group scoring significantly higher than the non-sports group; and there are significant group differences in the level of “academic performance” among them with the sports group scoring significantly higher than the non-sports group.

(2) There are significant differences in the levels of self-efficacy among college students of different genders. The general self-efficacy and self-regulation efficacy scores of female students were significantly higher than those of male students.

(3) Self-efficacy fully mediates between proactive personality and academic performance; the mediating role of self-efficacy between proactive personality and academic performance is non-significant across sport groups and genders.

## Data availability statement

The raw data supporting the conclusions of this article will be made available by the authors, without undue reservation.

## Ethics statement

Ethical review and approval was not required for the study on human participants in accordance with the local legislations and institutional requirements. Written informed consent for participation was not required for this study in accordance with the national legislation and the institutional requirements.

## Author contributions

XL designed and directed the project. ML and HY verified the analytical methods. ZZ performed testing and provided data. ZH helped supervise the project. All authors contributed to the article and approved the submitted version.
